# Self-Esteem in Women with Endometriosis: A Qualitative Study of Psychosocial Burden and Self-Esteem-Related Experiences

**DOI:** 10.3390/healthcare14152260

**Published:** 2026-07-24

**Authors:** Almut Bauder, Harald Werneck

**Affiliations:** Faculty of Psychology, University of Vienna, 1010 Vienna, Austria; almut.bauder@univie.ac.at

**Keywords:** endometriosis, self-esteem, medical invalidation, self-compassion, coping, psychosocial support

## Abstract

**Background/Objectives:** Endometriosis affects around 10% of women of reproductive age and is commonly associated with pelvic pain, fatigue and infertility. Beyond physical symptoms, the condition can substantially affect psychosocial well-being. However, self-esteem has typically been considered only indirectly in this context. This study examines how women with medically diagnosed endometriosis describe self-esteem in relation to the condition and the contexts in which it appears to be weakened or strengthened. **Methods:** Nine women with medically diagnosed endometriosis participated in semi-structured interviews (in person or online) between March and May 2025. Data were analyzed using Focused Interview Analysis with MAXQDA. **Results:** Self-esteem emerged as a recurring theme across everyday functioning, body-related experiences and, in some cases, fertility-related concerns. Self-esteem-related difficulties were described across different levels of symptom burden and were often discussed in relation to functional limitations, perceived reliability, loss of control, and high internal performance standards. Experiences of social and medical invalidation were frequently linked to self-doubt, withdrawal and self-invalidation. At the same time, participants described acceptance, self-compassion, boundary-setting, and the re-evaluation of femininity as experiences associated with a more stable sense of self-esteem. **Conclusions:** The findings suggest that self-esteem-related experiences were discussed in relation to functional limitations, experiences of being taken seriously, and available psychosocial resources. Self-esteem-related dynamics may represent a clinically relevant dimension to consider in psychosocial support for patients with endometriosis.

## 1. Introduction

Endometriosis is a benign, chronic gynecological condition characterized by the presence of endometrial-like tissue outside the uterus [1]. Symptoms are heterogeneous and do not necessarily correlate with the extent of the lesions [2]. Common symptoms include dysmenorrhea, dyspareunia, and chronic pelvic pain, accompanied by fatigue and other associated symptoms [2,3]. Overall, a complex, biopsychosocial clinical picture is described [4]. Despite sometimes severe symptoms, endometriosis often remains unrecognized initially. Due to their variability and overlap with other clinical presentations, the symptoms are frequently classified as nonspecific or attributed to other causes [2]. Furthermore, menstrual pain in particular is often considered “normal,” which can contribute to diagnostic delays of up to seven to ten years [1,2,5]. In addition to physical symptoms, endometriosis is associated with significantly reduced quality of life [6]. Chronic symptoms can have wide-ranging effects on psychosocial well-being and daily life, with quality of life being linked not only to pain burden but also to psychological distress and other psychosocial factors [7,8].

Women with endometriosis also frequently report experiences of invalidation, particularly before receiving a diagnosis, as their symptoms may be trivialized or attributed to psychological causes [9,10,11]. Such experiences have been associated with self-doubt, shame, guilt, and psychological distress [12,13,14]. More broadly, research on the psychosocial impact of endometriosis has primarily focused on quality of life and psychological distress [6,7,8], as well as body image concerns [15,16], fertility-related concerns [17] and changes in self-perception [18,19]. Self-esteem refers to an individual’s overall evaluative sense of personal worth and self-acceptance [20]. Although it may be related to psychological distress, body image, illness-related experiences, or quality of life, it represents a distinct construct concerned with how individuals evaluate themselves. However, self-esteem has rarely been examined as a central construct and has often been addressed only indirectly. Examining self-esteem more directly may therefore provide further insight into women’s psychosocial experiences of living with endometriosis.

Against this backdrop, the present qualitative study aimed to explore how women with endometriosis describe self-esteem-related experiences in the context of living with the disease.

## 2. Materials and Methods

The study employed a qualitative, exploratory design based on semi-structured interviews.

### 2.1. Study Design

To explore participants’ self-esteem-related experiences in depth, a qualitative, exploratory study design was chosen. Semi-structured interviews were used for data collection, as self-esteem is a subjective and context-dependent construct. This approach allowed participants to describe their experiences in their own words.

The goal was to gain a deeper understanding of subjective experiences, attributions of meaning, and self-esteem-related processes, rather than statistical representativeness.

### 2.2. Sample and Recruitment

We recruited women with medically diagnosed endometriosis. The participants identified as cisgender women and are therefore described as “women.” Additional information regarding the diagnosis was collected through self-report.

Recruitment took place via social media channels and newsletters from Austrian endometriosis organizations. Participants were recruited using a criterion-based purposive sampling strategy, with voluntary self-selection. Eligibility criteria were a self-reported medically confirmed diagnosis of endometriosis and willingness to participate in an interview.

The sample size was determined pragmatically within the scope and timeframe of this exploratory qualitative study. No formal assessment of data saturation or information power calculation was conducted. However, the focused research question, clearly defined eligibility criteria, and in-depth interviews allowed a focused exploration of self-esteem-related experiences within this specific sample.

A total of ten women participated; one interview could not be evaluated due to technical issues, resulting in a final sample of nine participants. Participants were recruited from Austria and Germany, ranged in age from 24 to 42 years, and had received their diagnosis one to six years prior. All participants were fluent German speakers. The majority reported having undergone laparoscopy and having prior experience with hormonal therapy. Details of the sample are summarized in Table 1 below.

### 2.3. Data Collection

The semi-structured interview guide was developed by the first author based on the research questions and the relevant literature. It provided a structured framework and included thematic sections on diagnosis and illness experiences, physical symptoms, psychological stress, body image, female identity, and self-esteem-related experiences. Each section included broad, open-ended main questions. The core topic areas were addressed across interviews to provide a common structure, while follow-up questions were used where appropriate to clarify or elaborate on participants’ accounts. Prior to data collection, the interview guide was pilot-tested in one interview that was not included in the final analysis. The full interview guide, including the main questions and follow-up prompts, is provided as Supplementary Material S1.

The interviews were conducted between March and May 2025 and took place either in person or online via Zoom. Six interviews were conducted in person and three online. All interviews were conducted in German by the first author, were audio-recorded, and lasted between 65 and 93 min. No prior relationship existed between the interviewer and participants. No other persons were present during the interviews, and no repeat interviews were conducted. All participants provided informed consent prior to participation.

### 2.4. Analysis

Data were analyzed using Focused Interview Analysis following Kuckartz and Rädiker [21], combining deductive category development based on the research questions with inductive refinement grounded in the interview material. The predefined interview domains informed the initial deductive coding framework but did not predetermine the final category system. The initial coding framework was organized into three broad domains: physical experiences, psychological experiences, and self-esteem-related experiences. Self-esteem was treated both as a distinct analytic domain and as a cross-cutting dimension within accounts of physical and psychological experiences. For analytic purposes, self-esteem was understood as participants’ evaluative relationship to themselves and their sense of personal worth. Psychological distress, body-image concerns, illness identity, perceived stigma, and reduced quality of life were not treated as indicators of self-esteem per se. Rather, they were considered self-esteem-related only when participants explicitly or implicitly linked these experiences to self-evaluation, self-worth, self-confidence, self-respect, self-blame, feelings of inadequacy, or worthlessness. After data collection, the interviews were transcribed and subsequently pseudonymized; personal details as well as specific references to locations or names were anonymized to protect the participants’ confidentiality. The transcripts were manually reviewed and corrected by comparing them with the audio recordings. The interviews were transcribed verbatim following Kuckartz and Rädiker [21]. The analysis was supported by MAXQDA (Version 26.0.0; VERBI Software GmbH, Berlin, Germany).

The transcripts were read multiple times to develop a deeper understanding of the participants’ accounts. Initial impressions and interpretive considerations were recorded in memos. Coding was performed iteratively, and we repeatedly reviewed, documented, and adjusted the category system throughout the analysis by adding, merging, or refining subcategories. In addition, memos and case summaries were created to capture central themes and connections within the individual interviews and to support both case-specific and cross-case interpretations. Recurring themes were identified by considering similarities and differences in coded passages across interviews, supported by analytic memos and case summaries. During the iterative refinement of the category system, related categories and recurring self-esteem-related experiences were grouped into four overarching themes for the presentation of the results. This grouping was guided by their relevance to the research question and their conceptual relatedness within the interview material. No formal saturation assessment was conducted.

### 2.5. Quality Criteria and Reflexivity

All transcripts were coded by the first author. No independent double-coding, formal assessment of coding agreement, or formal peer-debriefing procedure was undertaken. Coding consistency was supported through iterative re-reading of the transcripts, repeated review of coded material, and documentation of analytic decisions and adjustments to the coding system. Since self-esteem-related experiences were often not explicitly articulated but emerged in the context of physical and psychological experiences, the codes were repeatedly reviewed to avoid premature or overly interpretative conclusions. Personal assumptions and theoretical frameworks were continuously reflected upon during the analysis process.

## 3. Results

The analysis yielded four central themes: (1) functioning, performance norms, and self-esteem; (2) invalidation as a process linked to self-doubt and self-esteem; (3) self-esteem across body, femininity, and reproduction; and (4) potentially self-esteem-supportive experiences. Across interviews, these themes reflected recurring experiences as well as variation in participants’ accounts.

An overview of the main themes and illustrative quotations is provided in Table 2.

### 3.1. Functioning, Performance Norms, and Self-Esteem

Participants reported heterogeneous symptom burden, with current pain intensity ratings ranging from 1 to 9; some also reported cyclical peak values of 9. Self-esteem-related difficulties were described across different levels of symptom burden. Across interviews, these difficulties were primarily discussed in relation to functional limitations, perceived reliability, sense of control, and the ability to meet personal performance standards, rather than pain intensity alone.

Cycle-related lower abdominal pain, gastrointestinal symptoms, and fatigue were frequently mentioned. Seven participants described functional limitations in everyday life as a recurring source of self-esteem-related difficulties. Such limitations were often linked to feeling unable to “function normally,” having to cancel plans, or concerns about being perceived as unreliable.

In work-related contexts, several participants reported uncertainty about communicating their symptoms and concerns about possible negative reactions, for example: “How do I even communicate this? What do I do if they don’t react well?”

Alongside actual or anticipated external expectations, participants described high internal performance standards. Some reported trying to continue despite substantial symptoms, for instance by “pushing through” or being “pumped full of painkillers.” Self-esteem-related difficulties were particularly evident when participants felt unable to meet these expectations. As one participant explained: “Because the physical symptoms limit me so much that I can’t do so many things […] I often find myself having to cancel […] and then I automatically blame myself.”

Overall, physical symptoms were not automatically experienced as detrimental to self-esteem. Rather, they became self-esteem-relevant when participants experienced them as limiting everyday functioning or preventing them from meeting their own standards of performance and reliability.

### 3.2. Invalidation as a Process Linked to Self-Doubt and Self-Esteem

Across all interviews, participants reported experiences that they perceived as invalidating or dismissive of their own symptoms. Several participants characterized the diagnostic process as prolonged, often involving numerous doctor’s visits without a clear explanation of their symptoms. The diagnosis itself was experienced ambivalently. On the one hand, it was experienced as confirmation and relief, such as “then I was actually glad to have the diagnosis because I thought, I’m not crazy” or “it was just sudden confirmation that I hadn’t made it all up.” At the same time, the diagnosis was also perceived as burdensome, as it made the chronic nature of the illness apparent, for example, as “the best thing and at the same time the worst thing.”

Participants also identified gaps in knowledge as part of invalidating experiences, such as when symptoms were not taken seriously or when important information had to be sought out independently, for example, through other affected individuals online. Across interviews, invalidating experiences were not described solely as isolated events. Rather, several participants described a recurring process in which devaluation and trivialization were associated with self-doubt, self-invalidation, and, in some cases, withdrawal or silence.

Within medical settings, participants characterized invalidation as the dismissal of symptoms despite unremarkable test results. This was illustrated by statements such as “We’ve looked at everything; according to our readings, you’re a young, healthy woman.”

Trivialization emerged as a recurring pattern across both social and medical contexts. One participant recalled having heard for seven years: “That’s normal,” coupled with the message: “Well, you’re a woman, that’s just how it is,” and “you just have to put up with it.”

Several participants described menstrual-related stigmatization, particularly through the normalization of menstrual complaints. Examples included statements such as “Yeah, you’re just on your period” or “It’s part of being a woman; you just have to tough it out.” Such reactions were experienced as distressing and as conveying that participants’ symptoms were not being taken seriously.

Five participants also reported paternalistic communication during medical interactions. They described repeated recommendations for hormonal treatment and binary treatment framings, such as “either you have a child or you take the pill” or “either take the pill or have surgery.” One participant reported feeling like “one of 100” in this context and expressed a wish to be treated as an individual rather than being routinely prescribed hormonal treatment.

Some participants reported distressing discussions of more extensive procedures. One participant, who was 25 years old at the time, reacted to the prospect of “removing the uterus and ovaries” by stating: “That’s my uterus and my ovaries, not just anything.”

Three participants also described pronatalist framings in medical interactions, in which their symptoms or diagnosis were interpreted primarily in relation to pregnancy. One participant reported that the diagnosis “suddenly became important” when she wanted to get pregnant and “it wasn’t working.” Another described how her symptoms had repeatedly been narrowed down to pregnancy: “That must be your only concern.”

In addition to communication experiences, eight participants described hormonal treatment as stressful; one participant had not received hormonal treatment due to a pre-existing condition.

Participants described hormonal treatment as involving a difficult trade-off between physical symptom management and psychological well-being. Reported burdens included feeling “completely drained,” severe mood swings, and feeling unlike oneself. Some participants explicitly articulated the conflict between physical and mental health, for example: “If I don’t take the medication, I’m physically sick; if I do take it, I’m not doing well mentally—and that means I feel like I just have to decide: do I really want to be physically or mentally healthy?” One participant characterized the psychological burden associated with Ryeqo as feeling “like I’m being controlled by someone else,” adding: “That’s not me, that’s not me.” She further referred to the experience as “hell, an induced menopause in a young body” and stated: “The medication almost cost me my life, if I may put it that way, but unconsciously.” Several women also reported that they had deliberately discontinued hormonal treatments due to distressing side effects, such as: “No, I stopped everything completely—the hormones—because that was even worse.” Overall, hormonal treatment was commonly experienced as a difficult balancing process between physical relief and psychological strain.

Across interviews, experiences of trivialization and paternalistic communication in medical settings were frequently linked to self-doubt. One participant asked: “Am I really that stupid, or am I just imagining this?” Another stated: “You totally lose confidence in yourself.”

Several participants reported self-invalidation, that is, downplaying or questioning their symptoms. This included statements such as: “I’ve always had period cramps, and now it’s just worse,” as well as “Oh God, I’m just imagining this.” For some participants, self-invalidation took the form of an internal dialogue, for example: “Yeah, now you’re getting the period, now you’re going to throw up again. Yeah, you’re just imagining that […] I had to go to the bathroom all the time. Yeah, that’s because you’re imagining it.” One participant characterized this as a gradual process of self-deprecation and distancing from her own bodily perceptions:

You’re looking for an illness you don’t have. And that’s just kind of how it’s been, I’ve been shrinking more and more. And then I thought to myself, yeah, I’m actually not really listening to myself anymore, because if something hurts somewhere, I just tell myself, no, it can’t be that bad, they won’t find anything, there’s nothing wrong […] and then I basically completely sidelined myself as a person and ignored myself.

Explicit self-doubt was particularly evident following repeated dismissal of symptoms. Several participants described self-deprecation, loss of self-confidence, and feelings of not being good enough. In one account, repeated invalidation, in combination with self-esteem strongly linked to performance, was associated with increasing insecurity: “you just end up really doubting yourself […] about everything afterward,” leading to statements like “you’re actually worthless anyway.” Other participants similarly reported a loss of self-confidence and self-doubt in relationships and social contexts, for example: “I have absolutely no confidence in myself.”

For some participants, self-doubt was accompanied by increasingly withholding or downplaying symptoms over time. They described avoiding further discussions of their symptoms, particularly in medical contexts, because they feared being perceived as “overly sensitive.”

Silence also emerged as a way of avoiding anticipated judgment in everyday life. Participants reported mentioning symptoms only “relatively rarely,” minimizing them by saying, for example, “I just have a headache today, I can’t do it,” or withholding them for fear of being “judged for it”. In some cases, this was accompanied by social withdrawal, such as not wanting to go “out among people” and “pretend that I’m doing fine.”

### 3.3. Self-Esteem Across Body, Femininity, and Reproduction

Body-related self-esteem experiences were primarily situated within a tension between trust in one’s body, loss of control, and the evaluation of one’s own body. Several participants reported a conflict with their own bodies, accompanied by anger, frustration, and a loss of trust, for example, in the sense of “why is my body doing this to me?” or “what is my body doing?”

The body was sometimes experienced as unreliable or as a limitation, as illustrated by statements such as “I experience my body […] more as a limitation,” combined with comparisons like “Why do others have a functioning body and I don’t?”

Across interviews, body-related self-esteem difficulties were particularly evident when participants experienced their bodies as not functioning reliably. Visible bodily changes, including endo-belly and scars, were also experienced as distressing by some participants. For example, feeling “as if I were five months pregnant” was associated with insecurity and avoidance.

Self-esteem-related difficulties also emerged in relation to femininity and reproductive health. Several participants reported tensions in their experience of femininity, particularly in connection with cycle-related symptoms and the repeated awareness of reproductive organs. These tensions were reflected in statements such as “as if I weren’t a normal woman” or in the devaluation of femininity during difficult phases, even to the point of saying “I don’t want to be a woman anymore.” One participant summarized this as a recurring question: “Why does this have to be the case? Exactly. Just because I’m a woman.” Another participant reported having “demonized my femininity in many phases” and thought, “I don’t want to be a woman anymore.” A further participant stated, “I hate this organ.”

Fertility and the desire to have children were also relevant to self-esteem. This became apparent for some participants in fears of childlessness—such as “my greatest fear”—and self-blaming thoughts, such as “maybe it won’t work, and then it will be my fault.” Others rejected being reduced to their reproductive capacity, for example: “I’m not a living uterus that can be rented out.” At the same time, four participants explicitly reported no desire for children. For these participants, fertility-related concerns were often less central to their self-esteem, and they explicitly distanced themselves from societal expectations linking womanhood to motherhood. Participants emphasized that they did not define their value primarily through motherhood and expressed a clear differentiation from societal expectations in this regard. In some cases, this was also linked to concerns about passing on the illness. Additionally, internalized role expectations were evident in some accounts, such as “it would be my duty as a woman.” These expectations were often explicitly questioned or critically reflected upon.

At the same time, some participants reported re-evaluating femininity and distinguishing it from the illness. This was reflected, for example, in the view that pain did not define their identity, which was associated with a more stable sense of self-esteem. Participants emphasized that they were “not a lesser woman” and framed this as a form of self-respect: “I have respect, not only for other women, but also for myself.” Across interviews, the extent to which participants reported conflicts related to femininity varied, as did the extent to which they were able to integrate or positively re-evaluate their experience of womanhood.

Self-esteem issues also emerged in the context of sexuality, particularly in relation to pain, avoidance, and internalized expectations. Several participants described fear of pain and resulting reluctance regarding sexual activity, such as “I’m really hesitant about that now. Because I’m so afraid.” In some cases, this was accompanied by guilt and self-doubt, for example, “It’s my fault now” or “Am I actually a bad partner?”

### 3.4. Potentially Self-Esteem-Supportive Experiences

Seven participants described changes in how they related to themselves that were associated with a more stable sense of self-esteem. A key aspect involved linking self-esteem less strongly to performance and “functioning.” One participant reported no longer linking her “achievements so closely to [her] self-esteem” and increasingly “decoup[ling]” them from it. Another explained how fulfilling it was to accept herself “so unconditionally” and to trust “that others do the same.”

Supportive relationships were mentioned by all nine participants as an important social resource. These primarily referred to supportive relationships in their immediate circle, such as: “my partner, who has been through all of this with me for years.”

Seven participants identified self-compassion as central, involving a kinder approach toward one’s own body. One participant explained, “Because I had compassion for my body, we started working together again,” combined with the goal “so that we both feel good.” Olivia emphasized: “Actually, my body is so sick. […] I don’t need to be angry at it.” Self-acceptance was also reflected in statements such as: “I can be proud of myself” that “everything still works this way, despite the pain.”

Acceptance was likewise experienced as relieving. Participants reported allowing themselves to respond to bodily needs, for example: “I don’t care, and this is what my body needs right now” or “that’s just how it is now, I accept it.”

Five participants described setting boundaries as an important way of limiting external demands and responding more closely to their own needs. This included saying no at work “because I’m in pain” and the commitment to “stand up for myself unconditionally.” In one account, this more self-assured stance also extended to medical interactions. One participant reported how she now challenged explanations that did not fit her own experience: “And today I’m like, no way. So if a doctor tells me, ‘That’s not a side effect of XY,’ then I say, ‘Have you taken it? […] Are you a woman my age, with my health conditions—have you taken it? […] Because I’ve already been through that.’”

In some accounts, endometriosis was also framed as an opportunity for personal growth, including feeling “a little stronger” and “more resilient.” Two participants described psychotherapy as helping them separate pain from their self-image and their sense of womanhood. They associated this with greater self-acceptance and a more stable sense of self-esteem.

## 4. Discussion

This exploratory qualitative study examined how women with endometriosis described experiences related to self-esteem. In this small, context-specific sample, self-esteem-related difficulties were discussed not only in relation to symptom intensity, but also in relation to the functional meaning of symptoms, invalidating interactions, and experiences involving the body, femininity, sexuality, and reproduction. Participants also reported self-compassion, acceptance, supportive relationships, and boundary-setting as experiences associated with a more stable sense of self-esteem. These findings should be understood as preliminary and are discussed below in relation to the existing literature.

### 4.1. Functioning, Performance Norms, and Self-Esteem

Within this sample, self-esteem-related difficulties were not described solely in relation to symptom intensity. Rather, they became particularly salient when symptoms limited everyday functioning, reliability, or perceived control, and when these limitations conflicted with personal standards of performance. This finding is consistent with qualitative research indicating that unpredictable endometriosis symptoms may be experienced as a loss of control [22]. It can also be interpreted through the lens of contingent self-esteem, according to which self-worth may be especially vulnerable when it is closely tied to performance and competence [23].

Pain burden remains relevant to this interpretation, as it was frequently linked to reduced quality of life and perceived stress [24]. However, the present findings suggest that pain became self-esteem-relevant not only in terms of its intensity, but also in terms of how resulting limitations were understood and evaluated. This is in line with the literature describing chronic pain as a subjective and multifactorial experience influenced by cognitive, emotional, and social factors [25,26,27].

Facchin et al. [15] similarly found that psychological distress in women with endometriosis was related not only to pelvic pain, but also to individual factors including self-esteem, body image, and emotional self-efficacy. Previous research has likewise described illness-related limitations in endometriosis as being accompanied by frustration and feelings of guilt [28,29]. In the present study, such reactions appeared particularly relevant when symptom-related limitations were interpreted as a failure to meet personal standards of functioning and reliability.

Accounts of hormonal treatment further illustrated that symptom management could be experienced as a tension between physical relief and psychological well-being. In some accounts, declining or discontinuing hormonal treatment was associated with a greater sense of autonomy. Similar tensions surrounding treatment decisions have been described in qualitative studies [30,31].

### 4.2. Invalidation as a Process Linked to Self-Doubt and Self-Esteem

Participants’ accounts suggest that invalidating interactions in medical and social contexts were relevant to self-doubt and reduced trust in one’s own perceptions. Previous research likewise indicates that women with endometriosis are often not taken seriously, particularly before diagnosis, and that symptoms may be downplayed or trivialized [9,10]. In medical settings, complaints may also be prematurely attributed to psychological causes [11], which has been associated with self-doubt regarding one’s own pain experience [12]. Related experiences have been discussed in the literature under the term “medical gaslighting” [11].

Rather than being described solely as isolated negative events, invalidating experiences were, for some participants, embedded in a recurring process in which repeated devaluation was associated with self-doubt, self-invalidation, and, in some cases, silence or withdrawal. Similar qualitative findings suggest that invalidation may reinforce doubts about one’s own perceptions and contribute to concealing symptoms [9]. Such experiences have also been linked to shame, guilt, and the tendency to downplay one’s own symptoms [9,13,14].

Previous research has also identified stigma as a relevant factor in endometriosis-related psychological distress [14]. In particular, the normalization of menstrual pain and limited public and medical understanding of endometriosis may contribute to experiences of not being taken seriously [32,33].

The ambivalent experience of receiving a diagnosis, which combined relief and validation with confrontation with the chronic nature of the condition, is also consistent with earlier qualitative findings [9,34,35].

Taken together, these preliminary findings suggest that validating communication and greater awareness of endometriosis may be relevant not only for timely diagnosis, but also for reducing uncertainty and self-doubt among affected women.

### 4.3. Self-Esteem Across Body, Femininity, and Reproduction

Participants’ accounts suggested that body-related self-esteem difficulties were discussed primarily in relation to bodily unpredictability, reduced trust in the body, and illness-related limitations, rather than to appearance alone. Qualitative studies have similarly described body-image disruption in endometriosis, including feeling “not at home” in one’s own body and changes in affective and perceptual aspects of body image [19,31,36]. Visible changes, including endo belly and scars, were additionally described as unsettling in the present study. Previous research has linked body esteem and body-image concerns to psychological outcomes in endometriosis [15,16], while mixed-methods research on endo belly further highlights its relevance to body image and negative affect [37]. Although female identity remained generally stable among the participants, some reported tensions in their experience of femininity when cycle-related symptoms or societal expectations became particularly salient. Conversely, some participants distinguished pain and illness from their sense of womanhood and discussed this distinction in relation to a more stable sense of self-esteem. Research among childless women with endometriosis indicates that beliefs about motherhood, female identity, and infertility are associated with psychological health [17].

Fertility-related concerns also varied substantially across participants. For those with a strong desire to have children, possible infertility was described as self-esteem-relevant when concerns about childlessness were accompanied by guilt or perceived failure. In contrast, several participants without a desire for children explicitly rejected the idea that their value as women depended on motherhood. These differing accounts suggest that the self-esteem relevance of fertility varied according to individual values, life plans, and the personal meaning attached to motherhood. Pain-related sexual difficulties were also described as self-esteem-relevant when fear of pain was accompanied by guilt or concerns about being an inadequate partner. This observation is consistent with research linking body-image disturbance to sexual distress in individuals with endometriosis [38].

Taken together, the findings suggest that the self-esteem-related relevance of the body, femininity, sexuality, and reproduction varied according to the individual meanings participants attached to illness-related limitations, personal values, and life plans. Given the small qualitative sample, these observations should be interpreted as preliminary and should not be generalized to all women with endometriosis.

### 4.4. Potentially Self-Esteem-Supportive Experiences and Clinical Implications

Participants identified acceptance, self-compassion, supportive relationships, boundary-setting, and, in some cases, a re-evaluation of femininity as experiences associated with a more stable sense of self-esteem. These experiences coexisted with substantial illness-related difficulties. Qualitative research likewise suggests heterogeneous self-related experiences in endometriosis, including both psychosocial burden and disruptions in self-concept as well as self-acceptance, knowledge acquisition, and positive changes in self-perception [18,28]. In the present study, some participants reported a shift away from evaluating their worth primarily through performance and functioning toward a more accepting and compassionate relationship with themselves and their bodies. Previous research has likewise examined self-compassion and body-related self-perceptions in relation to psychosocial experiences among women with endometriosis [38,39].

In two accounts, participants characterized therapeutic support as helping them distinguish pain from their broader sense of self and advocate for their own needs. However, the present study was not designed to evaluate the effects of psychotherapy or other specific interventions. Participants’ wishes for earlier diagnosis, greater public awareness, and validating communication reflected the difficulties reported throughout the interviews. Taken together, these preliminary findings suggest that care that takes symptoms seriously and addresses psychosocial concerns alongside medical management may be valuable for women with endometriosis. Further research is needed to examine how such approaches can be implemented and whether they improve psychosocial and self-esteem-related outcomes.

### 4.5. Limitations and Future Research

A strength of this study lies in its in-depth qualitative approach, which enabled the exploration of experiences relevant to self-esteem that have often been addressed only indirectly in endometriosis research to date. At the same time, several limitations must be considered.

The small qualitative sample does not allow for statistical generalization. In addition, recruitment through endometriosis organizations and social media may have disproportionately reached women who were already more engaged with their condition or who had experienced substantial psychosocial burden, including invalidation. This potential self-selection may have contributed to the prominence of invalidation and medical dismissal in the findings. The findings must also be interpreted within the specific cultural and healthcare contexts of Austria and Germany. Moreover, as self-esteem is a broad and global construct, it cannot be determined to what extent the self-esteem-related experiences described were specifically attributable to endometriosis rather than to other psychological stressors. Furthermore, participants referred to different phases of illness and treatment, which limited a precise temporal classification of some experiences. It is also important to note that the findings do not permit conclusions about the intentions of healthcare professionals; rather, they reflect how participants experienced these interactions and the meanings they attributed to them.

Future research should examine whether the preliminary themes identified in this study are also found in larger and more diverse samples, across different healthcare settings, and at different stages of the disease course. Mixed-methods and quantitative research could further investigate associations between endometriosis-related experiences, self-esteem, and psychosocial distress while taking relevant contextual factors into account.

## 5. Conclusions

This exploratory qualitative study suggests that women with endometriosis may experience self-esteem-related difficulties not only in relation to symptoms themselves, but particularly when symptoms interfere with everyday functioning, challenge valued aspects of identity, or are met with invalidation. Participants also described acceptance, self-compassion, boundary-setting, and supportive relationships as experiences associated with a more stable sense of self-esteem. Given the small and context-specific qualitative sample, these findings are preliminary and do not establish causal relationships. Nevertheless, they underline the potential value of care that takes symptoms seriously and addresses psychosocial concerns alongside medical management. In particular, the frequently reported absence of validation highlights the importance of sensitive and respectful responses within both social and healthcare contexts. Participants also identified psychological support as a desired and potentially valuable resource for coping with stress, self-doubt, and illness-related limitations.

## Figures and Tables

**Table 1:** Sample characteristics of the nine participants included in the study. Demographic and clinical characteristics are summarized. ✓ indicates yes/present; and x indicates no/absent.

Pseudonym	Age	Year of Diagnosis	Diagnosis	Current Symptom Severity (Self-Reported, 1–10)	Current Hormonal Treatment	Desire for Children	Children
Sandra	27	2023	Laparoscopy	1	✓	Yes	x
Tamara	27	2020	Laparoscopy	7	x	Ambivalent	✓
Anna	26	2020	Laparoscopy	5	x	Yes	x
Chiara	27	2021	Laparoscopy	5–6	x	No	x
Olivia	42	2020	Laparoscopy	4–8	✓	No longer	✓
Lena	26	2019	Laparoscopy	4–9	x	No	x
Kathrin	24	2024	Laparoscopy	2	✓	Yes	x
Viktoria	29	2023	Ultrasound	1	✓	No	x
Britta	33	2024	Laparoscopy	3	x/Hormonal IUD	No	x

**Table 2:** Overview of main themes, core analytic findings, and illustrative quotations.

Main Theme	Core Analytic Finding	Illustrative Quotation
Functioning, performance norms, and self-esteem	Symptoms became self-esteem-relevant when they restricted everyday functioning and threatened participants’ sense of reliability or ability to meet personal standards.	“Because the physical symptoms limit me so much that I can’t do so many things […] I often find myself having to cancel […] and then I automatically blame myself.” (T)
Invalidation, self-doubt, and self-esteem	Repeated dismissal of symptoms was described in relation to self-doubt and questioning one’s own bodily perceptions.	“Am I really that stupid, or am I just imagining this?” (B)
Self-esteem across body, femininity, and reproduction	Bodily symptoms and reproductive experiences could become self-esteem-relevant when they challenged participants’ sense of womanhood and bodily functioning.	“Why does this have to be the case? Exactly. Just because I’m a woman.” (S)
Potentially self-esteem-supportive experiences	Self-compassion, acceptance, and boundary-setting were described as supporting a less performance-based relationship to self-worth.	“Actually, my body is so sick. […] I don’t need to be angry at it.” (O)

Note. Quotations were translated from German. Initials in parentheses refer to the participant pseudonyms used in Table 1.

## Data Availability

The data are not publicly available due to the sensitive nature of qualitative interview data and to protect participant confidentiality.

## Supplementary Materials

The following supporting information can be downloaded at: https://www.mdpi.com/article/10.3390/healthcare14152260/s1. Supplementary Material S1: Interview Guide; Supplementary Material S2: Participant Information and Consent Form.
