# Supplementary material for: Self-Esteem in Women with Endometriosis: A Qualitative Study of Psychosocial Burden and Self-Esteem-Related Experiences
Source: Healthcare (Basel). 2026 Jul 24;14(15):2260. doi: 10.3390/healthcare14152260 (PMC13465058; doi:10.3390/healthcare14152260)
Supplement: Supplementary file 1 [file healthcare-14-02260-s001.zip › Supplementary Material S1_Interview Guide.pdf]

The interview guide is presented in English alongside the original German version.

Supplementary Material S1: Halbstrukturierter Interviewleitfaden (Deutsch)

Einleitung

Danke, dass du dir heute die Zeit nimmst, mit mir über deine Erfahrungen und Erlebnisse zu sprechen. In dieser Studie möchte ich besser verstehen, wie Frauen mit Endometriose ihre Erkrankung erleben und welche Bedeutung diese für ihr persönliches Erleben haben kann. Endometriose betrifft weltweit etwa 10 % aller Frauen und obwohl die körperlichen Symptome weithin bekannt sind, gibt es nur wenige Forschungsergebnisse zu den psychischen Auswirkungen und wie diese das Selbstbild beeinflussen. Im Mittelpunkt des Interviews stehen deine persönlichen Erfahrungen mit der Erkrankung. Du kannst dir so viel Zeit nehmen, wie du möchtest, um auf die Fragen zu antworten. Wenn du mit einer Frage oder einem Thema nicht weitermachen möchtest, ist das völlig in Ordnung. Alle Antworten werden anonymisiert, sodass keine Rückschlüsse auf deine Identität gezogen werden können. Alles, was du heute teilst, wird vertraulich behandelt und ausschließlich für diese wissenschaftliche Untersuchung verwendet. Das Interview dauert ca. 60 Minuten und wird aufgezeichnet, um sicherzustellen, dass alle Informationen korrekt erfasst werden. Wenn das für dich in Ordnung ist, können wir beginnen.

Thema 1: Erfahrungen mit der Endometriose

Hauptfragen

- Wann hast du die Diagnose Endometriose erhalten?
- Wie hast du die Diagnose erlebt?
- Wie lange hat es von den ersten Symptomen bis zur Diagnose gedauert?
- Wie würdest du deine derzeitigen Beschwerden beschreiben?

Thema 2: Körperbild und Körpererleben

Hauptfrage

Wie würdest du dein Verhältnis zu deinem Körper im Zusammenhang mit der Endometriose beschreiben?

Mögliche Nachfragen

- Hat sich deine Wahrnehmung deines Körpers im Laufe der Zeit verändert?
- Gibt es Situationen, in denen du dich besonders wohl oder unwohl in deinem Körper fühlst?
- Hat die Erkrankung beeinflusst, wie du im Alltag mit deinem Körper umgehst (z. B. Kleidung, Berührungen oder Intimität)?
- Gibt es Situationen, in denen du dich deinem Körper besonders verbunden oder eher entfremdet fühlst?
- Kannst du diese Situationen näher beschreiben?

Thema 3: Physische und psychische Auswirkungen der Endometriose

Physische Erfahrungen

Hauptfrage

Welche körperlichen Beschwerden erlebst du im Zusammenhang mit der Endometriose und wie wirken sie sich auf deinen Alltag aus?

Mögliche Nachfragen

- Haben diese Beschwerden beeinflusst, wie du dich selbst wahrnimmst?
- Haben sie dein Vertrauen in deinen Körper oder in dich selbst verändert?
- Gibt es Situationen, in denen du dich aufgrund deiner Beschwerden anders als andere fühlst?
- Haben deine Beschwerden beeinflusst, wie du eigene oder äußere Erwartungen erlebst?

Psychische Erfahrungen

Hauptfrage

Wie erlebst du die psychischen oder emotionalen Auswirkungen der Endometriose?

Mögliche Nachfragen

- Welche Gefühle oder Belastungen sind für dich besonders bedeutsam?
- Haben diese Erfahrungen beeinflusst, wie du über dich selbst denkst?
- Haben sie dein Vertrauen in dich selbst oder dein Selbstbild verändert?
- Gibt es Situationen, in denen sich diese Erfahrungen auf deine Beziehungen oder den Umgang mit anderen Menschen auswirken?

Thema 4: Weiblichkeit und Identität

Hauptfrage

Hat die Endometriose beeinflusst, wie du dich selbst als Frau erlebst?

Mögliche Nachfragen

- Gibt es bestimmte Bereiche (z. B. Fruchtbarkeit, Sexualität, körperliche Erscheinung oder Menstruation), die dabei für dich eine besondere Bedeutung haben?
- Gab es bestimmte Erfahrungen, die deine Sicht auf dich selbst als Frau beeinflusst haben?
- Hat sich dein Verständnis von Weiblichkeit im Laufe der Zeit verändert?
- Falls ja: Wie würdest du diese Veränderung beschreiben?

Abschluss

- Gibt es etwas im Zusammenhang mit deiner Endometriose, das wir bisher noch nicht angesprochen haben und das dir wichtig erscheint?

- Was würdest du dir für die zukünftige Forschung oder den gesellschaftlichen Umgang mit Endometriose wünschen?

Vielen Dank, dass du deine Erfahrungen mit mir geteilt hast.

Zum Abschluss würde ich noch zwei kurze soziodemografische Angaben aufnehmen:

- Wie alt bist du?
- Wo ist dein aktueller Wohnort?

Supplementary Material S1: Semi-Structured Interview Guide (English Translation)

Introduction

Thank you for taking the time to speak with me today about your experiences and perspectives. This study aims to better understand how women living with endometriosis experience their condition and what meaning it may have in their lives. Endometriosis affects approximately 10% of women worldwide and although the physical symptoms are widely recognized, there is still limited research on the psychological effects of the condition and how these may influence self-perception. The interview will focus primarily on your personal experiences of living with endometriosis. You may take as much time as you need to answer the questions. If at any point you do not wish to continue with a question or topic, that is completely fine. All responses will be anonymized so that no conclusions can be drawn about your identity. Everything you share today will be treated confidentially and used exclusively for scientific research purposes. The interview will take approximately 60 minutes and will be audio-recorded to ensure that all information is captured accurately. If this is okay with you, we can begin.

Section 1: Experiences with Endometriosis

Main Questions

- When were you diagnosed with endometriosis?
- Can you tell me about your experience of receiving your diagnosis?
- How long did it take from your first symptoms until receiving a diagnosis?
- How would you describe your current symptoms?

Section 2: Body Image and Body Experience

Main Question

How would you describe your relationship with your body in the context of living with endometriosis?

Possible Prompts

- Has your perception of your body changed over time?
- Are there situations in which you feel particularly comfortable or uncomfortable in your body?
- Has endometriosis influenced how you relate to your body in everyday life (e.g., clothing, physical touch, or intimacy)?

- Have there been situations in which you felt particularly connected to or disconnected from your body?
- Could you describe these situations?

Section 3: Physical and Psychological Experiences

Physical Experiences

Main Question

Which physical symptoms of endometriosis are most relevant in your daily life, and how do they affect you?

Possible Prompts

- Have these symptoms influenced the way you see yourself?
- Have they affected your confidence in your body or yourself?
- Are there situations in which you feel different from others because of your symptoms?
- Have your symptoms influenced how you experience your own or other people's expectations?

Psychological Experiences

Main Question

How have the emotional or psychological aspects of living with endometriosis affected you?

Possible Prompts

- Which emotions or psychological challenges have been particularly important for you?
- Have these experiences influenced the way you think about yourself?
- Have they affected your confidence or self-image?
- Are there situations in which these experiences influence your relationships with other people?

Section 4: Femininity and Identity

Main Question

Has living with endometriosis influenced how you experience yourself as a woman?

Possible Prompts

- Are there particular aspects (e.g., fertility, sexuality, physical appearance, or menstruation) that have been especially meaningful for you?
- Have there been specific experiences that influenced how you see yourself as a woman?
- Has your understanding of femininity changed over time?
- If so, how would you describe this change?

Closing Questions

- Is there anything about your experience with endometriosis that we have not discussed but that you consider important?
- What would you like to see in future research or public awareness regarding endometriosis?

Thank you very much for sharing your experiences. Finally, I would like to ask two brief demographic questions:

- How old are you?
- Where do you currently live?
