# Supplementary material for: Self-Esteem in Women with Endometriosis: A Qualitative Study of Psychosocial Burden and Self-Esteem-Related Experiences
Source: Healthcare (Basel). 2026 Jul 24;14(15):2260. doi: 10.3390/healthcare14152260 (PMC13465058; doi:10.3390/healthcare14152260)
Supplement: Supplementary file 1 [file healthcare-14-02260-s001.zip › Supplementary Material S2_Participant Information and Consent Form.pdf]

Teilnehmerinneninformation und Einverständniserklärung

Studie:
Selbstwert bei Endometriose

Studienleitung:
Almut Christine Bauder
Fakultät für Psychologie, Universität Wien

Informationen zur Teilnahme:
Du bist eingeladen, an einer qualitativen Interviewstudie teilzunehmen, die untersucht, wie sich Endometriose auf Selbstwertgefühl, weibliches Identitätsgefühl und Körperbild auswirken kann. Im Mittelpunkt des Interviews stehen persönliche Erfahrungen im Leben mit Endometriose. Die Teilnahme an dieser Studie ist freiwillig. Du kannst einzelne Fragen unbeantwortet lassen oder das Interview jederzeit ohne Angabe von Gründen beenden, ohne dass dir daraus Nachteile entstehen. Das Interview dauert ungefähr 60 Minuten und wird aufgezeichnet, um eine korrekte Transkription und Auswertung sicherzustellen. Alle im Interview geteilten Informationen werden vertraulich behandelt. Personenbezogene Daten werden anonymisiert, sodass keine Rückschlüsse auf deine Identität möglich sind. Die erhobenen Daten werden ausschließlich für wissenschaftliche Forschungszwecke im Rahmen dieser Studie und damit verbundener wissenschaftlicher Veröffentlichungen verwendet.

Einverständniserklärung: Ich bestätige, dass ich die Informationen zur Studie gelesen und verstanden habe und freiwillig an der Studie teilnehme.

Name: ____________________________

Unterschrift: ______________________

Participant Information and Consent Form

Study:
Self-Esteem in Endometriosis

Principal Researcher:
Almut Christine Bauder
Faculty of Psychology, University of Vienna

Information About Participation:
You are invited to participate in a qualitative interview study exploring how endometriosis may affect self-esteem, female identity, and body image. The interview focuses on personal experiences related to living with endometriosis. Participation in this study is voluntary. You may decline to answer individual questions or stop the interview at any time without any negative consequences. The interview will take approximately 60 minutes and will be audio-recorded to ensure accurate transcription and analysis. All information shared during the interview will be treated confidentially. Personal data will be anonymized so that no conclusions can be drawn about your identity. The collected data will be used exclusively for scientific research purposes within the scope of this study and related scientific publications.

Consent: I confirm that I have read and understood the study information and voluntarily agree to participate in this study.

Name: ___________________________

Signature: ________________________
